# Effect of Traditional Drying Methods on Proximate Composition, Fatty Acid Profile, and Oil Oxidation of Fish Species Consumed in the Far‐North of Cameroon

**DOI:** 10.1002/gch2.202000007

**Published:** 2020-04-15

**Authors:** Noël Tenyang, Roger Ponka, Benard Tiencheu, Fabrice Tonfak Djikeng, Hilaire Macaire Womeni

**Affiliations:** ^1^ Department of Biological Sciences Faculty of Science University of Maroua P.O. Box 814 Maroua Cameroon; ^2^ Department of Agriculture Livestock and By‐Products National Advanced School of Engineering University of Maroua P.O. Box 46 Maroua Cameroon; ^3^ Department of Biochemistry Faculty of Science University of Buea P.O. Box 63 Buea Cameroon; ^4^ School of Agriculture and Natural Resources Catholic University Institute of Buea P.O. Box 563 Buea Cameroun; ^5^ Department of Biochemistry Faculty of Science University of Dschang P.O. Box 67 Dschang Cameroon

**Keywords:** fatty acids, food composition, freshwater fish, lipid oxidation

## Abstract

The purpose of this study is to evaluate the effects of two traditional drying methods on proximate composition, lipid oxidation, and fatty acid composition of two freshwater fish species from Maga Lake in Cameroon. As shown by the results, these two methods significantly (*p* < 0.05) decrease moisture content in the two fish species while ash and lipid content significantly increases (*p* < 0.05). The highest content of proteins is obtained for sun‐dried fish. Analysis also reveals that these two drying methods accelerate lipid oxidation by increasing peroxide value and total oxidation value. Smoking and sun drying decrease polyunsaturated fatty acid/saturated fatty acid and *n*‐3/*n*‐6 ratios of the two fish oils. The higher percentages of unsaturated fatty acid present in raw samples are responsible for the major changes in fatty acid profile occurring during drying methods. It is concluded that based on lipid oxidation, sun‐drying is found to be the better method to dry fish.

## Graphical abstract

Mullet (*Liza falcipins*) and carp fish (*Oreochromis niloticus*) from Maga Lake of Cameroon are a good source of proteins and lipids. During processing, lipid, protein, and ash contents increase. High ash in dried fish is in accordance with their high minerals content. Increase consumption of these fish can help to prevent many diseases to the consumers, and protein deficiencies.

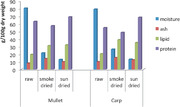

## 1 Introduction

For many centuries, fish has been one of the main foods for humans and constitutes an important part of diet in many countries. They are good sources of important nutrients and constitute desirable components of healthy diet. The high nutritional value and easy digestibility are the advantages of fish as food. Fishes are rich source of omega‐3 (*n*‐3) long chain polyunsaturated fatty acids (PUFAs). These are essential for maintaining the integrity of membrane of all living cells. PUFAs are responsible to produce prostaglandins which regulate inflammation and blood clotting. The fish also contains eicosapentaenoic acid (EPA) and docosahexaenoic acid (DHA) which serve as important components in the reduction of some risk factors associated with arteriosclerosis and heart disease.^[^ ^1^ ^]^ The *n*‐3 PUFAs are very important because they play a vital role in the development and functioning of nervous system (brain), photoreception (vision), and reproductive system.^[^ ^2^ ^]^ The fatty acid composition of fish may vary from one fish species to another and between freshwater fish to marine water fish, so it is important for human health, to increase the consumption of fish.^[^ ^3^ ^]^ Fish being an important source of animal proteins, play a significant role in the diet of many people in developing countries. Their amino acids are nutritionally superior to that of cereals grains. Fish protein can therefore be used to ameliorate protein quality of mixed diet.^[^ ^4^ ^]^ As a way of extending storage life and increasing its economic market value, fresh fish in Far North of Cameroon is processed through sun drying, smoking, and freezing.^[^ ^5^ ^]^ The preservation effect of smoking and sun drying are mainly due to the decrease in water activity and thus prevention of growth of many spoilage microorganisms.^[^ ^6^ ^]^

Mullet (*Liza falcipins*) and carp fish (*Oreochromis niloticus*) are the common fishes present in many lakes in Far‐North region in Cameroon. They were usually sold as a raw product. The application of smoking and sun drying for extension of the shelf‐life is a process of interest, given that these fishes are generally fatty fishes which spoil easily.

Several authors pointed out that the processing can affect the proximate composition, the lipid composition of fish, especially the fatty acids content, by changing the nutritional value of processed products in relation of raw samples. Moreover, Tenyang et al.^[^ ^7^ ^]^ reported that heat treatment can lead to undesirable changes, such as loss of essential fatty acids, reducing nutritional value of fish, mainly due to lipids oxidation. However, there is a great variability in changes concerning individual fatty acids in response to different processing methods.^[^ ^7^ ^]^

Despite the various studies focusing on the effect of processing methods on nutritive value, lipid oxidation and fatty acids profile of different fish species (e.g., refs. [7, 8, 9, 10, 11]), as far as we know, very limited information has been reported regarding the effect of processing on water fish species commercialized and consumed in Far North region of Cameroon. The main objective of this work was to investigate the effect of two traditional drying (smoking and sun drying) on proximate composition, lipid oxidation, and fatty acids profile of mullet (*L. falcipins*) and carp fish (*O. niloticus*) from Maga Lake of the Far North region in Cameroon. The scientific information obtained could serve as an important input for human nutrition and maximum utilization.

## 2 Experimental Section

### 2.1 Sampling and Preparation of Fish

The samples of 45 fresh raw fish for each species were bought from fish Maga market in the Far North region of Cameroon in the month of May. These fishes were from Maga Lake. This area is located between latitude 10° and 13° North and between longitude 13° and 16° East. Its climate is of the tropical Sudano‐Sahelian type with two seasons: a long dry season of about eight months, from October to May, and a short rainy season of four months, from June to September.^[^ ^12^ ^]^ The average weight of each fish was 250 g with lengths ranging from 20 to 30 cm. Once collected, they were immediately transported to the laboratory in iceboxes. Until arrival to the laboratory, the fishes were carefully washed with clean, cooled, and tap water. Scales and viscera were removed and again washed with clean tap water to remove blood. They were rinsed with distilled water. The fish samples were divided in three lots. The first was use as a control, the second and the third lot were prepared respectively for the sun drying and smoking process.

### 2.2 Drying Methods

#### 2.2.1 Sun Drying

For sun drying process, the cleaned mullet and carp were cut into two equal halves. Cut was making along longitudinal axis of the fish body from mouth to tail but the two halves of the body remained attached in the tail fin region. During sun drying procedure, the unsalted raw mullet and carp (eight fish samples by species) were dried by exposing to ambient sunlight at daytime (8 a.m to 5 p.m) for 3 days (due to the climatic conditions in drying period, moisture content of air was comparatively less [26%]). The average wind velocity was 13.30 km h^−1^. At the same time, temperature was recorded (25–47 °C). During sun drying, the fishes were covered with mosquito net to prevent insects and others pests. The fishes during the sundry processing were turned over from time to time to ensure homogenous drying. After drying, the sun‐dried samples were packaged with polythene plastic bag and stored in dry condition for further analysis.

#### 2.2.2 Smoking

For smoking process, the cleaned whole fresh mullet and carp were used. The fresh fish were spread out on smoking trays after washing without salting. The trays were then stacked on smoking oven fired with hard wood, and marked at temperatures greater than 70 °C. Fish were continuously smoked and to obtain a dried smoked fish, the process took 7 h. Fish samples during smoking were turned at intervals to ensure homogenous drying.^[^ ^7^ ^]^ Samples of fresh mullet and carp were homogenized and used as untreated control fish.

### 2.3 Lipid Extraction

After smoking and sun drying treatments, oils were extracted from raw and processed fish by the method described by Bligh and Dyer.^[^ ^13^ ^]^ About 80 g of fish were introduced in a grinding machine (Panasonic, Kyoto, Japan) to which 100 mL of chloroform and 200 mL of methanol were subsequently added. The mixture was grinded for 3 min; followed by the addition of 100 mL of chloroform and 100 mL of distilled water. The mixture was again grinded for 1 min, and filtered. The final extraction was ensured by the addition of chloroform, in the order to attain the following proportion: 2:2:1.8 for chloroform, methanol, and distilled water respectively. After separating the different phases in a funnel, the organic phase was collected and dried using sodium anhydrous. The organic solvent was then eliminated by evaporation on a rotator evaporator at 45 °C under reduced pressure. The extracted oil was stored at 4 °C for further analysis.

### 2.4 Analytical Methods

#### 2.4.1 Proximate Analysis of Fish

Moisture content of the fish samples was determined by thermal drying to constant weight at 110 °C for 12 h. Ash content was determined by burning sample for 12 h in a furnace at 550 °C. Crude protein content was determined by Kjeldahl analysis (nitrogen × 6.25; Kjeltec Autonalyser, Teaator, Hoganas, Sweden). Lipid content was determined using Soxhlet apparatus with hexane. All methods were based on those described in the Association of Official Analytical Chemists.^[^ ^14^ ^]^ All samples were analyzed in triplicate.

#### 2.4.2 Chemical Analyses of Fish Oil

##### Measurement of Free Fatty Acid

The determination of free fatty acid (FFA) of fish oil samples was made according to the method described by AFNOR.^[^ ^15^ ^]^ The results were expressed as % oleic acid.

##### Analysis of Iodine Value

The iodine value (IV) of fish oil samples was determined using the Wijs method, as described in the AOAC official method.^[^ ^16^ ^]^ The IV was expressed as g I_2_ per 100 g of sample.

##### Peroxide Value

The peroxide value was determined by referring to the IDF standard method, 74A: 1991.^[^ ^17^ ^]^ The results were expressed as milliequivalents of O_2_ per kg sample.

##### *p*‐Anisidine Value

Anisidine value was determined by the standard AOCS Cd 18–90 « *p*‐anisidine value » using a Perkin Elmer UV‐Visible Spectrophotometer (Norwalk CT, USA).^[^ ^18^ ^]^

##### Total Oxidation Value

Total oxidation (TOTOX) values of oil samples were determined using the equation TOTOX = 2PV + AV according to Shahidi and Wanasundara.^[^ ^19^ ^]^

#### 2.4.3 Fatty Acids Composition of Fish Oil

Oil extracted from the powdered fish was used for the determination of fatty acids profile. The lipids were transmethylated using NaOH/MeOH followed by BF_3_/MeOH according to the method described by Metcafe et al.^[^ ^20^ ^]^ The fatty acids methyl esters were analyzed on a Hewlett Packard 5880 gas chromatography (GC) equipped with a flame ionization detector. The esters were separated on a 50 m × 0.20 mm internal diameter wall coated open tubular fused silica capillary column coated with Carbowax 20M. Column injector and detector temperature were 200 and 300 °C, respectively. The carrier gas was helium and the split ratio was 100:1. Identification and quantification of fatty acids were performed by comparison of their peak with the relevant peak areas of the corresponding standard fatty acids. Each fatty acid was then expressed as a percentage of the total fatty acids quantified. All the experiments were carried out in triplicate.

#### 2.4.4 Polyene Index of Fish Oil

The polyene index used to determine lipid oxidation of fish oil during processing is a good indicator.^[^ ^21^ ^]^ It was calculated according to the following formula: PI = EPA + DHA/C16:0.

### 2.5 Statistical Analysis

Each determination was performed in triplicate. Data were expressed as mean ± standard derivation (SD). Statistics were performed using the Microsoft Office Excel program. Differences were evaluated by ANOVA using Statistical Package of Social Science (SPSS 16.0). Levels of statistical significance were *p* < 0.05.

## 3 Results and Discussion

### 3.1 Effect of Sun Drying and Smoking on Proximate Composition of Mullet and Carp Fish

**Table** **1** shows the results expressed as “mean ± SD” of the effects of traditional processing on the proximate composition of mullet and carp fish. Sun and smoking drying as can be seen from this table caused significant decrease (*p* < 0.05) in moisture content of the two samples and sun‐dried fish had the lowest value of moisture content (≈13%). In 2014, Akinwumi^[^ ^8^ ^]^ observed similar trend during the evaluation of smoking process on the nutritive value of African Mud catfish. The changes noted during processing are mainly related to the dehydration caused by heat. Decrease of moisture content can be also explained in terms of denaturation of sarcoplasmic and myofibrillar proteins and disruption of the muscle structure, which lead to a decreasing water holding capacity of the proteins fraction. Moisture content in the sun‐dried fish were within the 15% acceptable limit to prevent microbial deterioration,^[^ ^22^ ^]^ suggesting that sun‐dried fish could be stored for a long time. Between the raw fish species, no significant (*p* > 0.05) difference in ash content was noted. However, the total ash content during processing increased and smoked fish were found to have the higher value of ash content. Smoke‐dried mullet compared to smoke‐dried carp presented the higher ash content. Increase in ash content in fish can be explained by the reduction in moisture during processing. These results are in accordance with those of Adeyeye et al.,^[^ ^23^ ^]^ who demonstrated that, the ash content of fish during drying process increases. The lipids content of fish samples ranged between 19.48% and 38.32%, with significant variation in content between individual treatments. The highest values of lipids (35% and 38% respectively in mullet and carp) were found in smoked fish samples in comparison to lowest values (19.48% and 20.50% respectively in mullet and carp) found in the fresh fish samples. Increase in lipids content could also be due to the dehydration caused by high temperature during processing. The increase of lipids content during drying confirms the findings noted by Tenyang et al.^[^ ^24^ ^]^ The variation in lipids content during processing may be link to the process applied.

**Table 1:** Effect of dried methods on proximate composition of fish

Samples of fish	Moisture [% wet weight]	Ash [g per 100 g dry weight]	Lipid [g per 100 g dry weight]	Protein [g per 100 g dry weight]
Mullet				
Raw	80.51 ± 1.70^a^	8.12 ± 0.07^e^	19.48 ± 0.82^e^	63.00 ± 0.02^b^
Smoke‐dried	21.56 ± 1.47^c^	17.00 ± 0.60^b^	35.16 ± 1.11^b^	57.32 ± 1.24^c^
Sun‐dried	13.02 ± 1.28^d^	10.00 ± 0.65^d,e^	32.19 ± 0.90^c^	68.92 ± 1.04^a^
Carp				
Raw	79.18 ± 0.60^a^	10.06 ± 0.20^d^	20.50 ± 0.60^d^	54.73 ± 0.32^d^
Smoke‐dried	26.53 ± 0.54^b^	15.40 ± 0.45^a^	38.32 ± 1.58^a^	48.57 ± 0.64^e^
Sun‐dried	13.31 ± 1.71^d^	12.54 ± 0.77^c^	35.10 ± 0.68^b^	68.45 ± 0.19^a^

Mean values in the same column with different superscript letters are significantly different (*p* < 0.05).

The proteins content of processed fish samples varied between 48.57% and 68.92%. Sun‐dried sample had the highest value of proteins content, while smoked fish samples had the lowest value. No significant variation in proteins content was observed between sun‐dried fish samples. A significant increase in the proteins content in sun dried‐fish indicates that the proteins nitrogen in drying period is not going to lose; this is in agreement with the results noted by Tao and Linchum^[^ ^25^ ^]^ during sun drying of fishes. However, decrease of proteins content noted in this work collaborates with the finding noted by Akinwumi^[^ ^8^ ^]^ during smoking of catfish. It is known that reduction of proteins could be due to denaturation of some nitrogen‐containing compounds due to high temperature and smoking duration. The decrease of protein content also increase the loss of nutritional value of fish.

### 3.2 Effect of Sun and Smoke Drying on Fish Oil Qualities

#### 3.2.1 FFA of Oil Extracted from Dried Fish

A progressive increase in FFAs occurred during traditional sun and smoke drying of these two Maga Lake fish (**Table** **2**). As can be seen from this table, the FFAs differ marginally between raw and dried samples. Smoked samples compared to sun‐dried samples presented the higher value of FFAs, while raw samples had a lower value. Between smoked samples, smoked mullet was found to present the highest FFAs (8.48% oleic acid). Increase of FFAs during traditional drying suggests that triglycerides and phospholipids present in fish muscle were partially hydrolyzed. Hydrolysis of lipid may be due to enzymes activities and thermolysis action due to high temperature applied. The high FFAs in sun‐dried fish compared to smoked fish may be due to methods and drying duration. During sun drying, fish were exposed to free air and some lipase originated from microorganism contamination might have taken place. Fennema^[^ ^26^ ^]^ during treatments demonstrated that heat enhances hydrolysis of triglycerides present in food. Eymard et al.^[^ ^27^ ^]^ also reported that treatment increased the FFAs of horse mackerel minced fish. Azad et al.^[^ ^28^ ^]^ noted that, FFAs contents increased from 4.55% to 5.12% within 4 days of drying and then gradually increased up to 10 days of drying (6.86%). As quality specifications for crude fish oil, Bimbo^[^ ^29^ ^]^ suggested that the FFAs content should range between 2% and 5% oleic acid. The FFAs of all smoked samples were higher than 5% oleic acid. However, the formation of FFAs itself does not lead to nutritional losses. However, accumulation of FFAs in fish oil is undesirable, since it may lead to catalyzed secondary reaction such as increased susceptibility to oxidation and development of off‐flavor. FFAs are known to have negative effects on protein solubility and cause texture deterioration.

**Table 2:** Changes in acid, iodine, peroxide, *p*‐Anisidine, and TOTOX values of fish oil samples during processing

Samples	Acid value [% oleic acid]	Iodine value [g I_2_ per 100 g of oil]	Peroxide value [meq O_2_ per kg of oil]	*p*‐Anisidine value	TOTOX value
Mullet					
Fresh	1.91 ± 0.50^e^	95.27 ± 0.67^c^	7.82 ± 0.04^b^	nd	15.63 ± 0.08^c^
Sun‐dried	4.10 ± 0.17^c^	96.06 ± 0.45^bc^	7.90 ± 0.16^b^	nd	15.80 ± 0.32^c^
Smoke‐dried	8.49 ± 0.85^a^	92.89 ± 0.45^d^	1.91 ± 0.17^e^	16.20 ± 0.86^a^	20.03 ± 1.19^b^
Carp					
Fresh	2.53 ± 0.79^de^	100.34 ± 0.67^a^	4.19 ± 0.05^d^	nd	8.39 ± 0.10^e^
Sun‐dried	4.70 ± 0.12^c^	96.38 ± 0.45^b^	5.41 ± 0.10^c^	2.02 ± 0.15^c^	12.84 ± 0.35^d^
Smoke‐dried	5.97 ± 0.28^b^	87.65 ± 0.22^e^	9.90 ± 0.04^a^	6.43 ± 0.22^b^	26.23 ± 0.30^a^

Mean values in the same column with different superscript letters are significantly different (*p* < 0.05); nd: not detected.

#### 3.2.2 Iodine Value of Oil Extracted from Dried Fish

The iodine values of raw and traditional dried fish are presented in Table 2. The IV of raw samples varied from 95.27 to 100 g I_2_ per 100 g of oil. Mullet was found to present lowest value. The IV of raw mullet obtained in this work was lower than 78.56 g I_2_ per 100 g of oil noted by Tenyang et al.^[^ ^30^ ^]^ in the same species collected in the littoral region of Cameroon. The changes can be due to environment or diet of the fishes.^[^ ^31^ ^]^ The results showed that a decreasing trend in the IV are noted during processing and smoke‐dried samples compared to sun‐dried samples presented the high IV. Between smoked samples, smoked carp had the lowest IV (87.65 g I_2_ per 100 g of oil). The decrease of IV could be attributed to high temperature and fresh air, which facilitates oxidation of free unsaturated fatty acids present in fish oil. The decreases of IV noted in this study were in agreement with the increase of peroxide value shown in Table 2. According to the work of Tenyang et al.^[^ ^7^ ^]^, smoking can cause the oxidative rancidity of lipid resulting in a decrease of IV as well as a decrease in the nutritional value of fish.

#### 3.2.3 Peroxide Value of Oil Extracted from Dried Fish

The PV of raw and dried fish oil are illustrated in Table 2. As can be seen from the table, there was no difference in the PV of raw and sun‐dried mullet. The PV of oil from processed carp has increased drastically, whereas decrease in peroxide value from the oil of smoked mullet has been noticed. A significant increase (*p* < 0.05) in PV noted were probably due to increase of oxidation of oil caused by heating and fresh air.^[^ ^32^ ^]^ In contrast to smoking carp, the decreased PV of smoked mullet could be due to the rapid decomposition of initial hydroperoxides formed into volatile and non‐volatile products.^[^ ^33^ ^]^ Some researchers have found that dry processing has a negative effect on the lipids stability of fish.^[^ ^23^, ^34^ ^]^ The PVs obtained in this study were below the acceptable level of 10–20 meq O_2_ per kg of fish oil.^[^ ^35^ ^]^ Results of this parameter indicated that smoking compared to sun drying accelerates mostly the rate of lipids oxidation.

#### 3.2.4 The p‐Anisidine Value of Oil Extracted from Dried Fish

Table 2 shows the *p*‐Anisidine values (*p*‐AV) which illustrates the formation of secondary oxidation products in raw, sun–dried, and smoked fish sample. As can be seen in the table, raw and sun‐dried mullet, and raw carp did not contain detectable *p‐*Anisidine (0.25%). A lower value of *p*‐Anisidine (2.02) was obtained in sun‐dried carp while the higher value (16.20) was found in smoked mullet. The presence or the increase of p‐Anisidine in treated fish was probably due to the destruction of hydroperoxides during processing into secondary oxidation products especially nonvolatile carbonyls (2‐alkenal and 2,4‐alkadienal) in the later stages of lipid oxidation.^[^ ^34^ ^]^ As shown by Shahidi and Zhong^[^ ^36^ ^]^, a desirable *p*‐AV of oil is <4.0, with an upper limit of 6.0. The low *p*‐AV indicates the best quality of oil. Based on the results obtained, sun drying compared to smoke drying is the better method for drying fish.

#### 3.2.5 The TOTOX Value of Oil Extracted from Dried Fish

To estimate the quality of oil, the TOTOX value may be used. The values of TOTOX reflect the initial and later stages of the oil oxidation. The lower value of total oxidation indicates a higher quality of the oil.^[^ ^16^ ^]^ The TOTOX values of raw and dried fish are shown in Table 2. As compared to the raw samples, TOTOX value in carp samples increased with processing and smoke dried sample were found to have the higher value (26.23). In the case of mullet, no significant (*p* < 0.05) difference in TOTOX value was noted between raw and sun‐dried samples. However, during smoking, significant (*p* < 0.05) difference was observed. Health Canada^[^ ^37^ ^]^ suggested that the TOTOX value of 26 might be the maximum limit. According to ours results, the TOTOX value of all fish samples used were in acceptable limits.

### 3.3 Effect of Drying on Fatty Acids Profile of Fish Oil

The most important fatty acids of raw and processed mullet and carp are shown in **Tables** **3** and **4** respectively. In raw mullet (Table 3), saturated fatty acids (SFA) were the most abundant (35.34%), followed by polyunsaturated fatty acids (PUFAs) (33.78%) and monounsaturated fatty acids (MUFAs) (31.97%). Raw carp muscle contained 38.60% of SFA_S_, 31.97% of MUFAs, and 26.18% of PUFAs. The omega‐3 fatty acids content was dominated in all samples by linolenic acid (C18:3), EPA (C22:5), and DHA (C22:6). Linoleic acid (C18:2), eicosatrienoic acid (C20:3), and arachidonic acid (C20:5) were the most important omega‐6 fatty acids. The *n*‐3/*n*‐6 ratio of mullet and carp (1.14 and 0.60 respectively) are high when compared to that of silver catfish (0.29).^[^ ^38^ ^]^ The presence of DHA in mullet and carp fish from Maga Lake suggests that these fish species can have a healing effect to alleviate muscle pain and inflammation. DHA and EPA have be reported to have preventive effects on human coronary artery disease.^[^ ^1^ ^]^ Therefore, fish has been suggested as a key component for a healthy diet in human. Significant levels of EPA and DHA in fish species examined in current study indicate that these species can be used to supplement essential fatty acids in the human diet.

**Table 3:** Changes in fatty acid composition of mullet fish at different conditions

Fatty acid as % of total fatty acids	Raw	Sun‐dried	Smoke‐dried
C14.0, Myristic	2.17 ± 0.01^b^	3.19 ± 0.03^a^	2.01 ± 0.39^c^
C15:0, Pentadecanoic	1.66 ± 0.02^b^	1.74 ± 0.10^a^	0.75 ± 0.08^c^
C16:0, Palmitic	23.01 ± 0.01^b^	24.23 ± 0.02^a^	22.23 ± 0.09^c^
C17:0, Heptadecanoic	1.80 ± 0.00^b^	2.19 ± 0.03^a^	1.25 ± 0.02^c^
C18:0, Stearic	5.50 ± 0.05^b^	6.71 ± 0.07^a^	5.03 ± 0.03^c^
C20:0, Arachidique	0.73 ± 0.04^a^	0.74 ± 0.02^a^	0.38 ± 0.01^b^
C22:0, Behenic	0.35 ± 0.01^b^	0.47 ± 0.02^a^	0.22 ± 0.01^b^
C23:0, Tricosanoic	0.30 ± 0.02^c^	0.71 ± 0.05^a^	0.38 ± 0.02^b^
C24:0, Lignoceric	0.22 ± 0.01^b^	0.26 ± 0.01^a^	0.29 ± 0.02^a^
ΣSFA	35.34 ± 0.17^b^	39.53 ± 0.45^a^	32.54 ± 0.67^c^
C16:1, Palmitoleic	7.48 ± 0.07^b^	6.29 ± 0.10^c^	8.24 ± 0.09^a^
C17:1*n*, *cis*‐10 heptadecenoic	1.14 ± 0.02^b^	2.05 ± 0.05^a^	0.95 ± 0.04^b^
C18:1(*n*‐9), Oleic	20.50 ± 0.08^c^	22.59 ± 0.27^b^	24.05 ± 0.35^a^
C20:1, *cis*‐11 Eicosenoic	0.79 ± 0.05^c^	1.76 ± 0.03^b^	1.89 ± 0.01^a^
C22:1, Erucic	0.27 ± 0.02^b^	0.09 ± 0.01^c^	0.56 ± 0.03^a^
ΣMUFA	30.18 ± 0.24^c^	32.78 ± 0.91^b^	35.69 ± 0.43^a^
C8:2, Linoleic (ω‐6)	11.40 ± 0.06^a^	9.96 ± 0.11^b^	8.61 ± 0.07^c^
C18:3, ϒ‐linolenic (ω‐6)	0.81 ± 0.01^a^	0.70 ± 0.05^a^	0.61 ± 0.04^b^
C18:3, α‐linolenic (ω‐3)	13.10 ± 0.12^a^	9.96 ± 0.22^b^	8.07 ± 0.17^c^
C20:2, *cis*‐11, 14 Eicosadienoic	0.57 ± 0.03^a^	0.53 ± 0.04^a^	0.32 ± 0.05^b^
C20:3, *cis*‐8, 11, 14 Eicosatrienoic (ω‐6)	2.60 ± 0.10^a^	1.79 ± 0.09^b^	1.55 ± 0.11^c^
C20:4, Arachidonic (ω‐6)	1.35 ± 0.02^a^	1.02 ± 0.03^b^	0.89 ± 0.06^c^
C20:5, Eicosapentaenoic (EPA) (ω‐3)	0.70 ± 0.03^a^	0.26 ± 0.05^b^	0.19 ± 0.03^b^
C22:6, Docosahexaenoic (DHA) (ω‐3)	3.19 ± 0.09^a^	2.83 ± 0.12^b^	2.11 ± 0.10^c^
ΣPUFA	33.72 ± 0.46^a^	27.05 ± 0.71^b^	22.23 ± 0.63^c^
Σω‐3PUFA	16.99 ± 0.24^a^	13.05 ± 0.29^b^	10.37 ± 0.30^c^
Σω‐6PUFA	14.81 ± 0.19^a^	13.47 ± 0.38^b^	11.66 ± 0.28^c^
ω‐3:ω‐6	1.14	0.97	0.89
PUFA/SFA	0.95	0.68	0.68
PI	0.17	0.13	0.10

Mean values in the same line with different superscript letters are significantly different (*p* < 0.05).

**Table 4:** Changes in fatty acid composition of carp fish at different conditions

Fatty acid as % of total fatty acids	Raw	Sun‐dried	Smoke‐dried
C14.0, Myristic	3.0.1 ± 0.00^b^	4.29 ± 0.03^a^	2.75 ± 0.01^c^
C15:0, Pentadecanoic	1.65 ± 0.01^c^	1.80 ± 0.02^b^	2.44 ± 0.03^a^
C16:0, Palmitic	21.00 ± 0.04^b^	24.64 ± 0.10^a^	20.51 ± 0.06^c^
C17:0, Heptadecanoic	2.97 ± 0.01^b^	2.84 ± 0.01^c^	3.31 ± 0.02^a^
C18:0, Stearic	8.12 ± 0.05^a^	5.54 ± 0.08^c^	6.10 ± 0.09^b^
C20:0, Arachidique	0.43 ± 0.02^a^	0.54 ± 0.05^a^	0.35 ± 0.03^b^
C22:0, Behenic	0.28 ± 0.02^a^	0.23 ± 0.03^a^	0.30 ± 0.04^a^
C23:0, Tricosanoic	1.01 ± 0.02^a^	0.79 ± 0.04^b^	0.29 ± 0.05^c^
C24:0, Lignoceric	0.13 ± 0.01^b^	0.11 ± 0.01^b^	0.18 ± 0.02^a^
ΣSFA	38.60 ± 0.18^b^	42.82 ± 0.37^a^	36.23 ± 0.35^c^
C16:1, Palmitoleic	7.10 ± 0.08^c^	8.51 ± 0.12^b^	9.25 ± 0.09^a^
C17:1*n*, *cis*‐10 heptadecenoic	1.23 ± 0.02^b^	1.04 ± 0.01^c^	1.50 ± 0.03^a^
C18:1(*n*‐9), Oleic	23.04 ± 0.15^c^	26.82 ± 0.21^b^	27.64 ± 0.32^a^
C22:1, Erucic	0.60 ± 0.01^a^	0.07 ± 0.01^b^	0.08 ± 0.01^b^
ΣMUFA	31.97 ± 0.26^c^	36.94 ± 0.38^b^	39.12 ± 0.81^a^
C8:2, Linoleic (ω‐6)	13.13 ± 0.15^a^	11.28 ± 0.20^b^	9.91 ± 0.15^c^
C18:3, ϒ‐linolenic (ω‐6)	0.50 ± 0.01^a^	0.50 ± 0.02^a^	0.40 ± 0.04^b^
C18:3, α‐linolenic (ω‐3)	4.31 ± 0.15^a^	4.00 ± 0.09^a^	3.70 ± 0.03^b^
C20:2, *cis*‐11, 14 Eicosadienoic	0.39 ± 0.01^a^	0.41 ± 0.05^a^	0.27 ± 0.07^b^
C20:3, *cis*‐8, 11, 14 Eicosatrienoic (ω‐6)	1.02 ± 0.08^a^	1.07 ± 0.26^a^	0.24 ± 0.15^b^
C20:4, Arachidonic (ω‐6)	2.25 ± 0.05^a^	2.17 ± 0.21^a^	2.02 ± 0.07^a^
C20:5, Eicosapentaenoic (EPA) (ω‐3)	2.05 ± 0.01^a^	1.19 ± 0.01^b^	0.77 ± 0.08^c^
C22:6, Docosahexaenoic (DHA) (ω‐3)	2.53 ± 0.07^a^	1.75 ± 0.12^b^	1.66 ± 0.04^b^
ΣPUFA	26.18 ± 0.53^a^	22.37 ± 0.96^b^	18.93 ± 0.63^c^
Σω‐3PUFA	8.89 ± 0.32^a^	6.94 ± 0.22^b^	6.23 ± 0.25^c^
Σω‐6PUFA	14.65 ± 0.23^b^	21.96 ± 0.69^a^	12.57 ± 0.41^c^
ω‐3:ω‐6	0.60	0.32	0.49
PUFA/SFA	0.67	0.52	0.52
PI	0.22	0.12	0.12

Mean values in the same line with different superscript letters are significantly different (*p* < 0.05).

Sun drying and smoking affect the fatty acids profile of mullet and carp. In general sun‐drying in all fish species increase SFAs and MUFAs, while smoking decrease SFAs and increase MUFAs (Tables 3, 4). The results obtained also demonstrated that sun‐drying and smoking in all samples induced a significant (*p* < 0.05) reduction of PUFAs groups. After processing, sun‐dried and smoked mullet were found to have the highest DHA content compared to carp fish species. All smoked samples exhibited lower C18:3*n*‐3, C20:5*n*‐3, and C22:6*n*‐3 contents than raw samples. This result is similar to those obtained by Aubourg and Ugliano.^[^ ^39^ ^]^ Thus, decrease in PUFAs content in fish oil during processing potentially attributed to the structural and chemical changes induced in cells fish during exposition on sun and smoke. Sun and high temperature facilitate attack of double bonds of unsaturated fatty acids presents in fish oil, resulting in the oxidation of lipids and the decrease of nutritive value of fish oil.^[^ ^39^ ^]^

The PUFAs/SFAs ratio is used to estimate the nutritional quality of food lipids, and health guidelines have recommended that this ratio should above 0.4.^[^ ^40^ ^]^ In the present study, all treatments presented a PUFAs/SFAs ratio more than 0.4. Another important index of cardiovascular health is the *n*‐3/*n*‐6 ratio. This ratio is also strongly correlated with mortality caused by cancer and inflammatory and autoimmune diseases, and nutritional guidelines recommended this ratio to be more than 1:4.^[^ ^41^ ^]^ Smoking and sun drying mullet decreased significantly (*p* < 0.05) the ratio of *n*‐3/*n*‐6 and smoked sample presented the lower value of *n*‐3/*n*‐6 ratio. Both treated samples however showed ratios higher than 0.5.

Ultimately, the consumers are concerned about the benefit of dietary food to their heart. According to the relative content of particular groups of fatty acids, sun and smoke dried methods induced a decrease in polyene index (PI) of fish samples (Tables 3 and 4). The PI of raw mullet and carp (1.52 and 0.79 respectively) were higher than those of sun‐dried (1.26 and 0.38 respectively) and smoked dried (0.85 and 0.29 respectively). The findings of the present study suggest that decrease in PI during processing indicated that oxidation is taking place during these phases. These results agree with those obtained with the titrimetric method in this work.

## 4 Conclusion

The results of the present investigation showed that raw mullet and carp are a good source rich in proteins and lipids. These fishes contain mainly palmitic acid, oleic acid, and linoleic acid. They also contain EPA and DHA, which are important fatty acids. Smoking and sun drying method evaluated changed proximate composition, oxidation parameters, and fatty acids composition of mullet and carp fish. Changes in proximate composition were more prominent in smoked samples. Lipids and proteins content increased significantly in all sun and smoked fish samples, while moisture content was found to be reduced. The two methods used seem to increase the percentage of MUFAs (mainly C18:1*n*‐9), and decrease the relative proportion of PUFAs in fish oil. Regarding the nutritive fatty acid ratios, the data suggest that drying methods decrease the fish PUFA/SFA and *n*‐3/*n*‐6 ratios. However, sun‐drying could improve fish lipid quality in all samples compared to smoke‐drying.

## Conflict of Interest

The authors declare no conflict of interest.
